# Cycling back to folate metabolism in cancer

**DOI:** 10.1038/s43018-024-00739-8

**Published:** 2024-05-02

**Authors:** Younghwan Lee, Karen H Vousden, Marc Hennequart

**Affiliations:** The Francis Crick Institute, 1 Midland Road, London NW1 1AT, UK

## Abstract

Metabolic changes contribute to cancer initiation and progression through effects on cancer cells, the tumour microenvironment and whole-body metabolism. Alterations in serine metabolism and the control of one-carbon cycles have emerged as critical for the development of many tumour types. In this Review, we focus on the mitochondrial folate cycle. We discuss recent evidence that in addition to supporting nucleotide synthesis, mitochondrial folate metabolism also contributes to metastasis through support of antioxidant defence, mitochondrial protein synthesis and the overflow of excess formate. These observations offer potential therapeutic opportunities, including the modulation of formate metabolism through dietary interventions and the use of circulating folate cycle metabolites as biomarkers for cancer detection.

## Introduction

The observation that cancer cells show increased nutrient uptake and alterations in metabolism was noted by Otto Warburg almost a century ago^1^. Since then, alterations in many metabolic pathways have been shown to underpin the acquisition of malignant behaviour, highlighting numerous potential options for therapeutic intervention^2^. The first – and arguably still the most clinically impactful – metabolic therapy was pioneered in the 1950’s by Sidney Farber, who used the folate analogue, aminopterin^3^, to induce remission of childhood acute lymphocytic leukaemia (ALL). The original rationale for this approach was based on Farber’s empirical observation that folic acid stimulated the progression of ALL. Since then, the anti-folates have been shown to function as competitive inhibitors of dihydrofolate reductase (DHFR), the enzyme that produces tetrahydrofolate (THF) from folate. THF is an essential cofactor in nucleotide synthesis^4^ and the inhibition of DHFR results in depletion of intracellular pools of folate species^5^, leading to reduced synthesis of purines and pyrimidines^6^. Today, inhibitors of folate metabolism such as methotrexate are used extensively as first line chemotherapy for many types of cancer^7^. However, antifolate drugs are accompanied by many adverse effects due to the requirement of folate in normal, non-malignant cells – leading to a search for strategies beyond direct inhibition of DHFR that retain the efficacy of anti-folates without the limiting toxicities.

**Figure 1:**
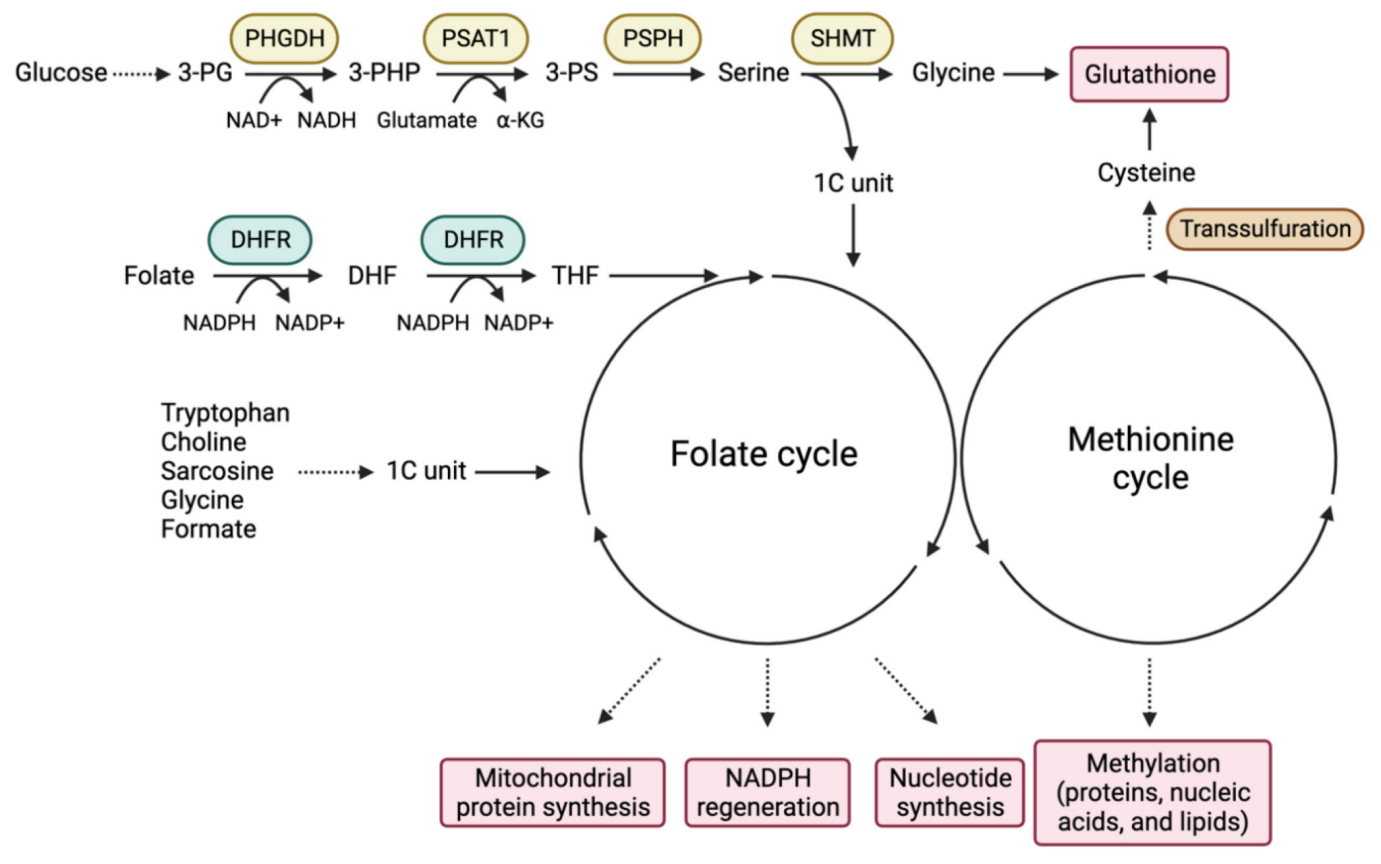
The serine synthesis pathway, folate cycle and methionine cycle. The serine synthesis pathway (SSP) uses glycolytic intermediate 3-phosphoglycerate (3-PG) in three sequential reactions to generate serine. First, phosphoglycerate dehydrogenase (PHGDH) catalyses the NAD^+^-dependent oxidation of 3-PG to 3-phosphohydroxypyruvate (3-PHP). Second, phosphoserine aminotransferase 1 (PSAT1) converts 3-PHP into 3-phosphoserine (3-PS) in a glutamate-dependent transamination reaction. Third, phosphoserine phosphatase (PSPH) generates serine through hydrolysis of 3-PS. Serine hydroxymethyltransferase (SHMT) converts serine into glycine by donating a one-carbon (1C) unit to tetrahydrofolate (THF). Glycine functions as a precursor for glutathione. Folate is reduced to dihydrofolate (DHF) and THF through dihydrofolate reductase (DHFR)-mediated NADPH-dependent reactions. THF serves as a carrier of 1C unit through the folate cycle. The folate cycle contributes to mitochondrial protein synthesis, NADPH regeneration and nucleotide synthesis. The folate cycle is coupled with the methionine cycle that generates the universal methyl-donor SAM for the methylation of proteins, nucleic acids and lipids and provides cysteine, a precursor of glutathione. Tryptophan, choline, sarcosine, glycine and formate can all generate 1C units.

Folate metabolism is part of the broader network of one-carbon (1C) metabolism, which describes the pathways that transfer 1C units for numerous physiological processes, such as nucleotide synthesis, methylation reactions, and redox defence. The 1C units that sustain the intersecting folate and methionine cycles are derived from several sources, including serine and glycine, tryptophan, choline and sarcosine metabolism (Figure 1). The methionine cycle is critically important for the production of S-adenosyl methionine (SAM), used for methylation reactions that modify and regulate nucleic acids, proteins and lipids, as well as the production of phospholipids, polyamines and cysteine (Figure 1). As such, the methionine cycle impacts all aspects of normal and diseased states, including cancer^8,9^. However, in this Review we will focus on the closely linked folate cycle, and recent advances in our understanding of its function in tumorigenesis.

While targeting folate metabolism to impair nucleotide synthesis is a well-established therapeutic paradigm, the folate cycle contributes to a wide range of metabolic reactions beyond nucleotide synthesis. As we will discuss, recent studies have revealed how the folate cycle can influence cancer progression and the tumour microenvironment through regulation of antioxidant defence, mitochondrial translation and formate overflow, revealing contributions of folate cycle intermediates on tumour growth, metastatic dissemination and the microenvironment.

### Serine and glycine metabolism

**Figure 2:**
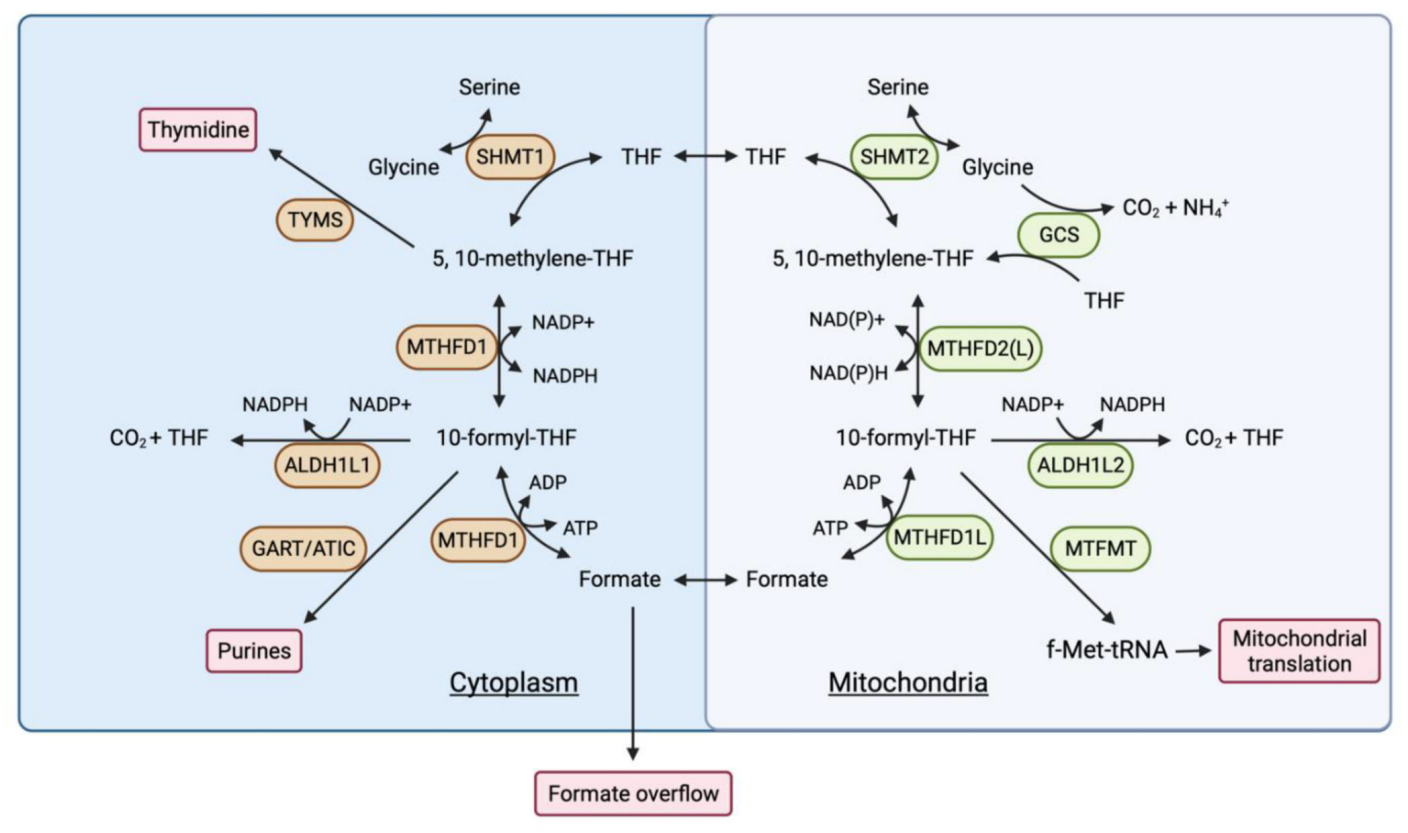
Folate metabolism in cytoplasm and mitochondria. Folate metabolism occurs in both the cytoplasm and mitochondria. Serine hydroxymethyltransferase 1 (SHMT1) in the cytoplasm or SHMT2 in the mitochondria generates glycine and donates one-carbon (1C) unit to THF, producing 5,10-methylene-THF. In the mitochondria, methylenetetrahydrofolate dehydrogenase 2 (MTHFD2) or MTHFD2-like (MTHFD2L) oxidize 5,10-methylene-THF to 5,10-methenyl-THF and 10-formyl-THF, regenerating NADPH or NADH. Mitochondrial 10-formyl-THF functions as the substrate for the generation of formate by MTHFD1-like (MTHFD1L), formylation of the methionine loaded mitochondrial initiator tRNA by mitochondrial methionyl-tRNA formyltransferase (MTFMT) for mitochondrial translation^18,19^ and the release of the 1C unit as CO_2_ by aldehyde dehydrogenase 1 family member L2 (ALDH1L2) in a reaction that regenerates NADPH. Mitochondrial formate is transported to the cytoplasm and used to regenerate 10-formyl-THF and 5,10-methylene-THF by MTHFD1. Surplus formate is excreted from the cell in a process termed formate overflow. The glycine cleavage system (GCS) can donate 1C units to the folate cycle. In the cytoplasm, cytosolic 5,10-methylene-THF serves as the substrate for thymidylate synthesis by thymidylate synthetase (TYMS). Cytosolic 10-formyl-THF functions as the substrate for the synthesis of purine nucleotides by phosphoribosylglycinamide formyltransferase (GART) and 5-aminoimidazole-4-carboxamide ribonucleotide formyltransferase/IMP cyclohydrolase (ATIC).

Serine and glycine are freely interconvertible, non-essential amino acid (NEAA) that support several metabolic pathways, including the production of phospholipids such as sphingolipids and phosphatidylserine, glutathione, porphyrins, creatine, purines and pyrimidines (Figure 2). However, serine is also a major donor of 1C units to the folate cycle. Serine can be taken up from the environment using several different transporters^10^ or generated through the *de novo* serine synthesis pathway (SSP). Briefly, the glycolytic intermediate 3-phosphoglycerate (3-PG) is converted to serine by three consecutive enzymatic reactions that are catalysed by phosphoglycerate dehydrogenase (PHGDH), phosphoserine aminotransferase 1 (PSAT1), and phosphoserine phosphatase (PSPH)^11^ (Figure 1).

Folate metabolism takes place in both the cytosol and mitochondria, using distinct but similar enzymes localised to each subcellular compartment (Figure 2). Mammalian cells are unable to produce folate (also called vitamin B9), which is present in standard culture media *in vitro* or provided in the diet or by the microbiota *in vivo*. Folate is reduced to tetrahydrofolate (THF – the biologically active form of folate) and dihydrofolate through an NADPH-dependent reaction catalysed by DHFR (the target of methotrexate). The conversion of serine to glycine by serine hydroxymethyltransferase 1 (SHMT1) in the cytosol or SHMT2 in the mitochondria produces glycine and donates a 1C unit to THF, producing 5,10-methylene-THF.

Interestingly, two recent studies have shown that the major source of folate in physiological circulation is 5-methyl THF, with unmetabolized folic acid contributing less than 10% of total folate species^12,13^. Dietary folates, mainly found in vegetables, exist mostly under the polyglutamyl form, which is cleaved into the corresponding monoglutamyl forms by glutamate carboxypeptidase II (GCPII) present at the brush-border of enterocytes. The monoglutamyl forms - predominantly 5-methylTHF - are then absorbed by the enterocytes and delivered into the circulation^14,15^. Cells taking up this form of folate utilise the vitamin B12 (cobalamin)-dependent enzyme methionine synthase (MTR) to transfer the methyl group from 5-methylTHF to homocysteine - producing methionine and supplying THF to the folate cycle^16,17^. This pathway for the acquisition of folate renders tumour cells less dependent on DHFR - and therefore resistant to methotrexate - but more sensitive to loss of MTR, which leads to folate trapping and reduction of purine and pyrimidine synthesis^16,17^.

In the mitochondria, 5,10-methylene-THF is oxidized by methylenetetrahydrofolate dehydrogenase 2 (MTHFD2) or MTHFD2-like (MTHFD2L) in a two-step reaction, the first producing 5,10-methenyl-THF – a reaction that also generates NADPH (or NADH) - and the second to produce 10-formyl-THF. Mitochondrial 10-formyl-THF serves as the substrate for three further reactions; the generation of formate by MTHFD1-like (MTHFD1L), formylation of the mitochondrial initiator tRNA by mitochondrial methionyl-tRNA formyltransferase (MTFMT)^18,19^, and the release of the 1C unit as CO_2_ by aldehyde dehydrogenase 1 family member L2 (ALDH1L2) – a second step in mitochondrial 1C metabolism to generate NADPH. A similar series of cytosolic enzymes also have the potential to generate 10-formyl THF. However, in most cells, the directionality of 1C metabolism is from the mitochondria to cytoplasm^20,21^. As 1C loaded THF conjugates cannot be transported across the mitochondrial membrane, mitochondrial formate is transported to the cytosol, where it serves to regenerate formyl-THF pools for purine synthesis and methylene-THF pools for thymidine synthesis, or is excreted from the cell in a process termed formate overflow^22,23^.

Interestingly, despite the predominance of the mitochondrial serine metabolism in supplying 1C unit for nucleotide synthesis, the cytosolic 1C flux from serine can compensate to sustain tumour growth when the mitochondrial pathway is inhibited^20^. However, ablating mitochondrial 1C metabolism can result in folate degradation in the cytosol, an effect that is also seen in response to methotrexate^21^. Overall, it has been suggested that the two connected cycles in the mitochondria and cytosol allow for the maintenance of both glycine and 10-formyl/ 5,10-methylene-THF synthesis^20^.

### Alternative sources of 1C units

While serine is the major donor of 1C units to fuel the folate cycle, other potential sources of 1C units can be found^12^. Both the production of glycine through choline metabolism and catabolism of glycine can result in the transfer of 1C units onto THF to produce 5,10-methylene-THF^24^. Glycine donates a 1C unit to THF to produce 5,10-methylene-THF in a reaction catalysed by a mitochondrial enzymic complex called the glycine cleavage system (GCS)^25^. However, the principal role of the GCS appears to be to limit glycine accumulation, with germline mutations in GLDC (a major component of the GCS) resulting in glycine encephalopathy^26^. Most recently, the 1C units produced by GCS have been suggested to drive reverse flux of SHMT2, producing serine and clearing excess mitochondrial glycine in the liver^27^. Choline contributes 1C units to support both the methionine cycle and – through the production of sarcosine – the mitochondrial folate cycle. Histidine catabolism can also produce 5,10-methylene-THF through the intermediate of 5-formimino-THF^28,29^ although the contribution of histidine degradation to the pool of 10-formyl-THF remains to be fully quantified.

Several other pathways sustain the 1C pool in cells through the production of formate. The catabolism of tryptophan produces N-formyl-kynurenine, which subsequently releases formate to form kynurenine. Formate produced through this pathway was shown to contribute to *de novo* purine synthesis and formate overflow^30^. Formate can also be directly produced by demethylation reactions, notably during cholesterol biosynthesis where lanosterol is demethylated to cholestatrienol by lanosterol 14α-demethylase^31^. The demethylation of endogenous metabolites or xenobiotics, and methanol metabolism also produce formaldehyde, a toxin that is either directly oxidized to formate by the mitochondrial NAD^+^ dependent aldehyde dehydrogenase (ALDH) 2 or indirectly through a detoxification pathway involving reduced glutathione and ALDH5^32,33^. Interestingly, formate generation from formaldehyde is sufficient to sustain the growth of cells that cannot utilize serine^33^. Finally, formate can also be produced and utilized by numerous bacterial species found in the colonic microbiome^34–37^.

### Alterations of serine & 1C metabolisms in cancer

Upon amino acid deprivation, eukaryotic cells activate an adaptive response called the integrated stress response to maintain cellular homeostasis^38^. This leads to the increased expression of the transcription factor activating transcription factor 4 (ATF4), which induces expression of serine and 1C metabolism-related genes, such as PHGDH, PSAT1, PSPH and SHMT2^39–42^. Serine starvation therefore upregulates expression of SSP genes to compensate for the loss of extracellular serine^40,43^. Activation of mammalian target of rapamycin complex1 (mTORC1), a major metabolic regulator, or induction of nuclear factor erythroid 2-related factor 2 (NRF2), a major antioxidant transcription factor, both also promote expression of SSP enzymes through ATF4^39,41,42,44^. Serine starvation activated ATF4 also induces the transcription factor ATF3, which in turn drives expression of the SSP enzymes^45^.

Serine itself can also modulate central carbon metabolism, by binding to and activating the M2 isoform of pyruvate kinase (PKM2) a glycolytic enzyme that controls flux of pyruvate to lactate. Decreased serine levels lower PKM2 activity, allowing increased channelling of glucose-derived carbon to de novo SSP^40,46^.

#### Genetic alteration in cancer

The importance of serine as a precursor of diverse pathways that control biosynthesis, epigenetic regulation, redox homeostasis, ATP generation, and TCA cycle regulation explains why cancer cells – and many normal cells – have a high demand for either endogenous or exogenous serine^47–49^. Consequently, genetic alterations of the SSP and 1C metabolism genes are frequently observed in cancers..

Copy number gain or overexpression of PHGDH and PSPH are found in several cancer types, including ER-negative breast cancers, melanoma, lung adenocarcinoma, T-cell ALL and osteosarcoma^48,50^. Chromosomal regions 1p12 and 7p11 - containing PHGDH and PSPH respectively - are amplified in cancers, with 7p11 amplifications potentially also encompassing the closely located oncogene, epidermal growth factor receptor (EGFR)^48,51–53^. In many cases, increased expression of the SSP enzymes correlates with poor prognosis^54–57^. In contrast to amplification, 5-methylthioadenosine phosphorylase (MTAP) - an enzyme that contributes to methionine salvage from S-adenosylmethionine (SAM) - is frequently deleted in cancer patients due to its proximity to the tumour suppressor gene, cyclin dependent kinase inhibitor 2A (CDKN2A), on chromosome 9p21^58,59^. Loss of MTAP leads to the accumulation of MTA and renders these cancer cells vulnerable to inhibition of protein arginine methyltransferase 5 (PRMT5).

Most cells can take up exogenous serine and in normal tissue loss of PHGDH (leading to loss of *de novo* serine synthesis) is generally well tolerated, although some changes in liver ceramide and lipid profiles are detected^60^. Interestingly, cancer cells can become dependent on PHGDH activity, even when supplied with exogenous serine, suggesting that serine uptake cannot always compensate from endogenously produced serine or that PHGDH has functions beyond serine synthesis (see below). By contrast, cells can become dependent on exogenous serine when decreased NAD^+^/NADH ratios impede the use of the SSP^47^. This was recently confirmed in mitochondrial malate/aspartate shuttle (MAS) deficient cells in which lower cytosolic NAD+/NADH ratios were accompanied by lower *de novo* serine synthesis. Supplementation of pyruvate restored the availability of NAD+ in MAS deficient cells, resulting in increased PHGDH activity^61^.

Downstream of serine, enzymes of the folate cycle are also found to be upregulated in multiple cancers. For example, RNA profiling of metabolic enzymes across 19 cancer types found MTHFD2 is frequently overexpressed ^62^. Enhanced expression of mitochondrial folate cycle enzymes such as SHMT2, MTHFD2, and MTHFD1L was also detected in cancer cell lines using metabolic profiling, and this was associated with higher mortality in breast cancer patients^63^. In glioma, increased SHMT2 expression was found to be critical for cancer cell survival and adaptation to the ischaemic tumour microenvironment (TME)^64^, a response that made cancer cells dependent on glycine decarboxylase (GLDC) to prevent the accumulation of excess glycine. The SHMT2 gene is amplified in B cell lymphomas due to copy number gain, contributing to lymphoma development by epigenetic modulation of tumour suppressor gene expression^9^. Progression to metastatic disease has also been linked to increased expression of mitochondrial serine and 1C metabolism genes^65^.

Enhanced expression of the GCS enzyme GLDC is detected in various cancers^63,66^, although this reflects a requirement of cancer cells to remove glycine rather than a need for 1C units. Interestingly, *in vitro* studies showed that in several cancer cell lines, glycine could not substitute for serine in supporting cell growth – rather increased glycine in the absence of serine inhibited proliferation^67^. In hepatocellular carcinoma (HCC), the GCS glycine cleavage system also plays a role in maintaining protein lipoylation and mitochondrial activity, rather than supplying 1C units^68^. Most recently, the 1C units produced by GCS have been suggested to drive reverse flux of SHMT2 to clear excess mitochondrial glycine in the healthy liver. This regulation of homeostatic glycine levels could be an important factor when dietary depletion of serine is considered for cancer therapy^27^.

#### Oncogene and tumour suppressor mediated control of serine metabolism

Reflecting the importance of serine metabolism in cancer development, several oncogenes and tumour suppressor have been shown to regulate serine and formate metabolism. MYC, a transcription factor frequently deregulated in cancers, increases the expression of SSP and mitochondrial 1C metabolism related enzymes, alone or in collaboration with other transcription factors such as activating transcription factor 4 (ATF4) or hypoxia-inducible factor1α (HIF1α)^69–72^. Activation of the internal tandem duplication of FMS-like tyrosine kinase 3 (FLT3-ITD) - an alteration frequently observed in acute myeloid leukaemia patients - also upregulates the SSP in a mechanistic target of rapamycin complex 1 (mTORC1)-ATF4-dependent manner, making these tumours sensitive to serine biosynthesis inhibition^73^. Similarly, inhibitor of nuclear factor kappa B (IkB) kinase ε, a regulator of cytokine secretion, can induce the SSP enzymes through ATF4 activation^74^. Mitochondrial respiratory chain defects – a common feature of malignancies^75^ – result in decreased flux through the mitochondrial 1C cycle but also lead to an ATF4 -dependent upregulation of SSP enzymes^76^. Activated KRAS increases the dependence of cancer cells on folate metabolism and concurrently upregulates expression of SSP enzymes through nuclear factor erythroid 2-related factor 2 (NRF2)^39,49,77,78^. NRF2 responds to oxidative stress and its ability to enhance serine metabolism has been linked to an increased production of NADPH for antioxidant defense^39^. In a pancreas cancer model, loss of liver kinase B1 (LKB1) with KRAS activation induced serine and 1C metabolism, altering the epigenetic landscape by upregulating SAM levels and DNA methylation^44^.

p53 is a tumour suppressor protein that is lost or mutated in most cancers^79^. p53 responds to oncogenic stress and - depending on the context - either helps cells to survive and adapt to mild or transient stress or eliminates cells exposed to persistent or severe stress^80^. Consistently, p53 can both support cell survival under conditions of serine starvation^81^ and sensitize cells to serine starvation by downregulating the expression of PHGDH^82^. The ubiquitin ligase, mouse double minute 2 (MDM2), is a transcriptional target of p53 that functions as an oncogene - in part by restraining p53 activity. MDM2 inhibits DHFR by catalysing its monoubiquitination^83^, but can also be recruited to chromatin to enhance serine and 1C metabolism independently of p53^84^.

Another key regulator of metabolic homeostasis that can function to suppress or support tumorigenesis is AMP-activated protein kinase (AMPK). AMPK indirectly downregulates expression of the 1C metabolism enzymes, MTHFD1, MTHFD1L and MTHFD2 in breast cancer, raising the possibility that AMPK activators may sensitize cancers to anti-folate therapy^85^. Atypical isoforms of protein kinase C (PKC) also regulate serine and 1C metabolism. PKCζ represses the expression of SSP enzymes, PHGDH and PSAT1 and inhibits PHGDH enzymatic activity by phosphorylation. PKCζ loss therefore promotes the ability of cancer cells to synthesize serine^86^. Additionally, loss of PKCλι induces serine metabolism through the mTORC1/ATF4-mediated mechanism, which contributes to neuroendocrine prostate cancer progression via increased DNA methylation^87^.

### How does serine and 1C metabolism support cancer development and progression?

The observation that many cancers depend on enhanced serine and 1C metabolism prompts the question of how they support tumorigenesis. Uncontrolled proliferation of cancer cells fuels a demand for nucleotide synthesis that can be met – in part – by enhanced 1C metabolism^67,88^, the importance of which has been well described and reviewed^89,90^. For example, increased mitochondrial 1C metabolism induced by the growth regulating kinase mTORC1 – which is frequently activated in cancer - is required for purine production and cell growth^42^. Upregulation of serine and 1C metabolism confers therapeutic resistance to 5-fluorouracil (5-FU), a commonly used chemotherapy that inhibits DNA synthesis^91^. As a result, 5-FU-resistant colorectal cancer cells become dependent on serine metabolism to support purine biosynthesis^92^, highlighting a potentially targetable induced vulnerability.

Despite the clear importance of nucleotide production, it is becoming increasingly clear that other aspects of serine, glycine and folate metabolism may contribute to cancer development, progression and metastasis.

#### Redox defence

**Figure 3:**
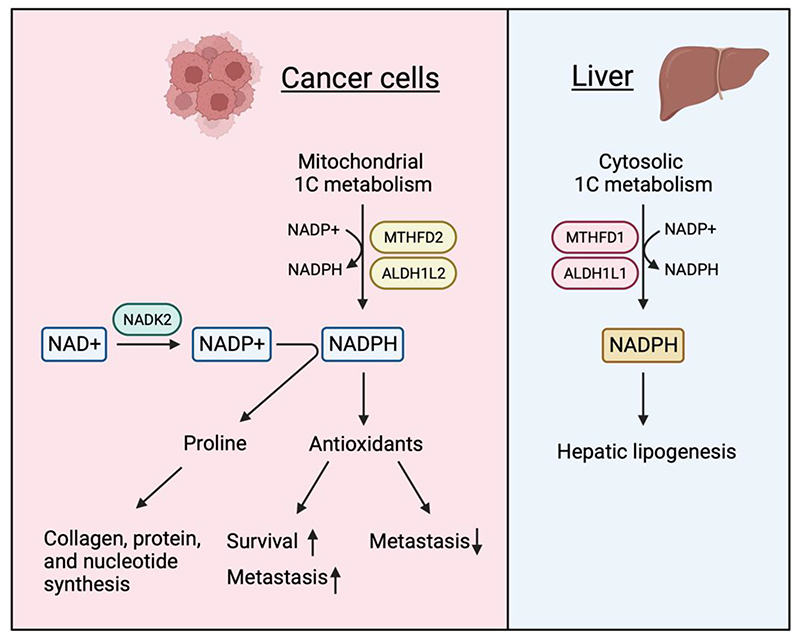
Mitochondrial and cytosolic NADPH use in cancer cells and hepatocytes. Cancer cells generate mitochondrial NADPH through methylenetetrahydrofolate dehydrogenase 2 (MTHFD2) and aldehyde dehydrogenase 1 family member L2 (ALDH1L2)-mediate mitochondrial one-carbon (1C) metabolism. Mitochondrial NAD kinase 2 (NADK2) produces NADP^+^ from NAD^+^, leading to an increase in the pool of NADPH. Increased NADPH pools in cancer cells are required for proline synthesis that supports the synthesis of collagen, protein and nucleotide and for antioxidant defence. Limitation of reactive oxygen species (ROS) can promote or impede metastasis in a context-dependent manner. In the liver, cytosolic NADPH generated through MTHFD1 and ALDH1L2-mediated 1C metabolism contributes to hepatic lipogenesis.

Serine and 1C metabolism can contribute to redox balance by either providing intermediates such as glycine and cysteine or by contributing to reactions that generate NADPH. Serine-derived glycine (from the SHMT reaction) and cysteine (from the transsulfuration pathway (Figure 1) are components of glutathione (GSH) - a major antioxidant molecule. Under limiting serine conditions, cancer cells prioritize the synthesis of GSH to maintain antioxidant defense^81^. 1C metabolism also generates NADPH, which is used to reduce the oxidized forms of GSH and thioredoxin – another antioxidant. As described above, a number of reactions in the 1C cycle use or generate NADPH, depending on the directionality of cycle^12^. Under most conditions, flow through the mitochondrial 1C pathway generates mitochondrial NADPH through the activity of MTHFD2 and ALDH1L2 (Figure 2). Perturbation of mitochondrial 1C metabolism decreases the NADPH/NADP^+^ and GSH/GSSG ratios^93,94^ and numerous studies have underpinned the role of serine metabolism in antioxidant defence in different contexts^72,94–98^. This activity becomes important during tumorigenesis, where oncogene activation, loss of normal stromal support and hypoxia lead to increased levels of reactive oxygen species (ROS) that result in a requirement for enhanced antioxidant activity to maintain viability (Figure 3). This requirement can be met by increased 1C metabolism, for example, hypoxia due to insufficient blood supply leads to the upregulation of SHMT2, leading to the maintenance of NADPH production and redox balance in multiple cancer cell lines^72,99^. In melanoma, inhibition of folate metabolism with methotrexate or ALDH1L2 knockdown decreases NADPH production and increases ROS^100^. Similarly, ALDH1L2 depletion has been shown to increase mitochondrial ROS levels in breast cancer cells^20,94,97,100,101^.

The upregulation of 1C metabolism-mediated antioxidant defence may also contribute to therapy resistance^102^. However, there are other pathways for mitochondrial NADPH regeneration (such as malic enzyme 2/3 and isocitrate dehydrogenase 2) or production (such as NAD kinase). Furthermore, NADPH itself is important for other functions, such as proline or lipid production^95,98,103,104^. How cells respond to the modulation of NADPH production through 1C metabolism is therefore highly dependent on the system and context.

#### Mitochondria translation

Serine-derived sphingolipids such as ceramides are required for mitochondrial function and cell death induced by serine starvation may be partly due to structural defects of the mitochondria mediated by a reduction in ceramide. Serine and mitochondrial 1C metabolism also make important contributions to mitochondrial protein production^105^. MTFMT uses 10-formyl-THF to formylate methionine loaded tRNA (fMet-tRNA), which initiates mitochondrial protein synthesis^18^. Deletion of SHMT2 in mice reduces the availability of fMet-tRNA, leading to a decrease in the production of mitochondrial proteins and defects in mitochondrial respiration^106^ that result in a failure of cancer cells to adapt under glucose starvation^19^. Interestingly, the conversion of 10-formyl-THF to THF and CO_2_ by ALDH1L2 not only produces NADPH, but also limits other 10-formyl-THF fuelled reactions, including the production of formyl-methionine (fMet) and formate^101^ (Figure 2). As a result, while deletion of ALDH1L2 in breast cancer cell lines led to more ROS, it also increased mitochondrial protein synthesis^101^.

#### Formate overflow

**Figure 4:**
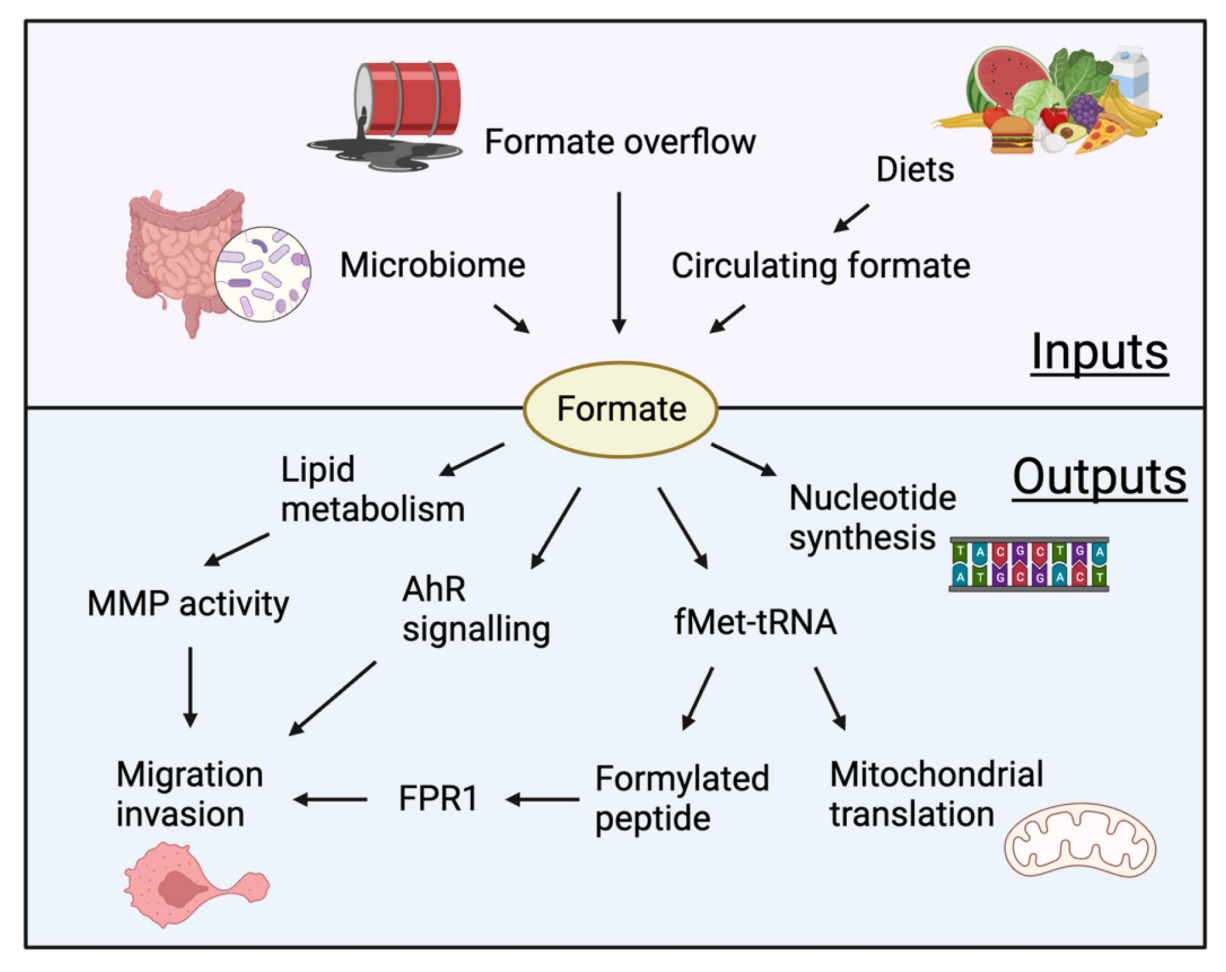
Inputs and outputs of formate metabolism. Formate can be derived from the microbiome, diet and formate overflow and can contribute to the behaviour of cancer cells through several mechanisms. Formate supports proliferation by functioning as substrate of nucleotide synthesis. Formate-derived formylated methionine-loaded tRNA (fMet-tRNA) initiates mitochondrial protein translation and migration and formylated peptides drive the invasion of cancer cells through formyl-peptide receptor (FPR) signalling. In addition, formate can increase migration and invasion of cancer cells through aryl hydrocarbon receptor (AhR) signalling and lipid metabolism/ matrix metalloproteinase (MMP) activity.

Formate is a key product of mitochondrial 1C metabolism, illustrated by the observation that the embryonic lethality resulting from deletion of MTHFD1L or the impaired T-cell function in aging mice that is associated with reduced mitochondrial metabolism can be at least partially rescued by formate supplementation^107,108^. Intriguingly, the rate of folate metabolism in normal cells and tissue exceeds the demand of 1C units for nucleotide synthesis, leading to an excess production of formate, which is excreted from the cell. Cancer cells show an even higher rate of formate overflow^23^, contributing to an increase in circulating formate in tumour bearing mice that is dependent on serine^23,109^ (Figure 4). However, circulating formate levels can be either increased or decreased in cancer patients, depending on the tumour type^110^. While serine starvation and inhibition of 1C metabolism enzymes such as MTHFD1L and MTHFD2 decrease formate production, depletion of ALDH1L2 allows for an increased use of 10-formyl-THF for formate production^101^. Several studies show an important role for formate overflow in supporting malignancies, with multiple explanations for the benefit of this apparently wasteful metabolic activity. Firstly, formate overflow may simply be a by-product of a high rate of flux through the mitochondrial 1C cycle to generate ATP and contribute NADH to support mitochondrial respiration. There is evidence that inhibiting MTHFD1L (the step producing formate from 10-formyl-THF) leads to a switch in energy production to glycolysis^23^ and, consistently, formate overflow was shown to be a hallmark of oxidative cancers, with formate levels directly correlating to NADH/NAD^+^ ratios in a large panel of cancer cells types^109^. Additionally, there is accumulating evidence that formate itself provides several advantages to cancer cells. Formate may induce a metabolic switch to favour nucleotide and energy production^111^, with formate availability leading to increased purine production, while formate deficiency results in a reduction of AICAR, an intermediate of *de novo* purine synthesis and activator of AMPK^111^.

In the context of cancer, the contribution of the formate dependent metabolic switch to cancer development could explain, to some extent, the dependency of these cancers on serine and 1C enzymes. Furthermore, recent studies have highlighted growth-independent consequences of formate overflow and have uncovered an ability of formate to increase the migrative and invasive potential of cancer cells, contributing to metastasis as discussed further below ^101,112^.

### A role for 1C metabolism intermediates in metastasis

Cancer mortality predominantly results from metastatic spread of the primary cancer to other organs, a point at which therapeutic interventions show limited efficacy. Disseminating cancer cells face numerous obstacles as they move out of the tumour mass, circulate in the blood or lymphatic system and enter distant organs to re-establish growth. One key requirement for metastasis is the ability to move into and out of the circulation (intravasation and extravasation), partially reflected in model systems by the ability to migrate and invade. A second requirement is the ability to survive increased oxidative stress that accompanies the changing environment.

Several studies have shown a link between availability of exogenous and endogenously produced serine and migration. For example, production of SSP derived α-ketoglutarate (α-KG) was shown to be responsible for enhanced breast cancer metastasis^113^. Interestingly, the pro-tumorigenic roles of PHGDH are balanced by the observation that loss of PHGDH also enhanced metastasis, in this case by promoting aberrant protein glycosylation^114^. Consequently, heterogeneity of PHGDH expression is linked to enhanced aggressiveness in primary cancers. Numerous other studies have implicated mitochondrial 1C metabolism activity in the metastatic process. Inhibition of purine synthesis was shown to accelerate cell migration by increasing serine synthesis and 1C metabolism, despite reducing proliferation^115^. Furthermore, hypoxia induced activation of PHGDH and 1C cycle enzymes supported the maintenance of metastasis-inducing breast cancer stem cells (BCSCs)^116^. Aggressive breast cancer cell lines also show upregulation of mitochondrial serine and 1C metabolism and in patients, expression of SHMT2 is correlated with poor survival^65^. Indeed, a common theme emerging from these studies is the observation that enhanced SSP and folate cycle activity can promote metastasis without impacting cancer cell proliferation or the growth of the primary tumour^101,112,113^.

These observations raise the question of how folate metabolism supports metastasis. Evidence is now emerging that control of ROS, formate production and mitochondrial protein synthesis may all play a role. The production of mitochondrial NADPH and consequent protection from excessive mitochondrial ROS have been shown to be critical in allowing metastasis of some tumour types (Figure 3). For example, the PHGDH-dependent protection of BCSCs mentioned above is linked to a switch to 1C cycle supported mitochondrial antioxidant defence^116^. Loss of the NADPH-producing enzyme ALDH1L2 leads to increased ROS and a decrease in tumour sphere formation of glioblastoma cells^117^ and metastatic capacity of melanoma cells^100^. However, the consequences of loss of antioxidant capacity can be context dependent, with increased ROS also promoting enhanced metastasis in some cancers^118^.

Formate is emerging as a potentially important regulator of metastasis, correlating with the high levels of formate overflow seen in many cancers (Figure 4). Exogenous formate can directly enhance migration and invasion of different cancer cell lines^101,109^ and a reduction of formate overflow in response to MTHFD1L silencing reduces migration *in vitro* and the incidence of lung metastasis in breast cancer models^109,112^. As mentioned above, ALDH1L2 expression can shift the balance between NADPH, formate and fMet production^101^, with decreased ALDH1L2 expression leading to lower NADPH production (and more ROS) but enhanced formate and fMet synthesis. In some models, both increased ROS and decreased formate/ fMet production contributed to increased cell migration and enhanced metastasis. Increased formate or decreased ALDH1L2 activity both resulted in an increase in the production of formylated peptides – produced from proteins initiated with fMet. A better-known source of formylated peptides are bacterial proteins (also initiated by fMet), which bind to the formyl-peptide receptors expressed on immune cells to promote immune cell migration and anti-bacterial defense^119–121^. Interestingly, cancer cells also express formyl-peptide receptors and the increased migration of these cells in response to formate or ALDH1L2 depletion reflects – at least in part – the production of formylated peptides and activation of formyl-peptide receptor signalling^101^. Expression of ALDH1L2 in tumours is variable, and while increased ALDH1L2 can promote metastasis by limiting mitochondrial ROS, in other cancers, metastases showed lower ALDH1L2 expression compared to the primary tumour, suggesting that in some cases there is a selection for enhanced formate production during dissemination. However, additional mechanisms through which formate functions have been described in other cancer types. For example, microbiome-derived formate can activate AHR to support colorectal cancer stemness and migration^34^. Formate has also been shown to favour migration of glioblastoma cells lines through upregulation of matrix metalloproteinase 2 (MMP2). In this case, depletion of MTHFD1L – which decreases formate levels - led to reduced MMP2 activity. Additionally, formate supplementation can increase the expression of genes implicated in lipid metabolism, resulting in reduced invasion of glioblastoma cells and decreased levels of MMP2 in response to fatty acid synthesis inhibitors. Targeting lipid metabolism may therefore provide a mechanism to limit the effect of formate overflow on migration^122^.

Taken together these studies highlight the role of formate in promoting invasion but also show that formate availability can be sustained by many different mechanisms. More work is needed to disentangle the role of each pathway that contributes to formate overflow and the different consequences of this phenomenon.

### Interplay between cancer cells and the TME

It is well established that the normal stromal cells in the TME play a key role in supporting the genesis and progression of tumours, including supporting the nutritional demands of tumour cells^123^. For example, while increased activity of the SSP can support tumour growth in conditions of low serine^124^, other cells in the TME – such as neurons - can also provide serine to the tumour^125^. Cancer cells themselves can induce or enhance the supportive role of most cell types in their periphery^126^ to support their survival and progression^126^. Tumour mediated stress signals trigger the induction of PHGDH in endothelial cells that drives a shift in central metabolism, triggering proliferation and vessel sprouting of the tumour associated endothelial cells that promote tumour growth^127^. Tumours also affect their microenvironment by excreting metabolites into the tumour interstitial fluid (TIF). TIF contains nutrient levels distinct from those found in the circulation^128^, with evidence for depletion or increase in specific nutrients depending on tumour type and location.

**Figure 5:**
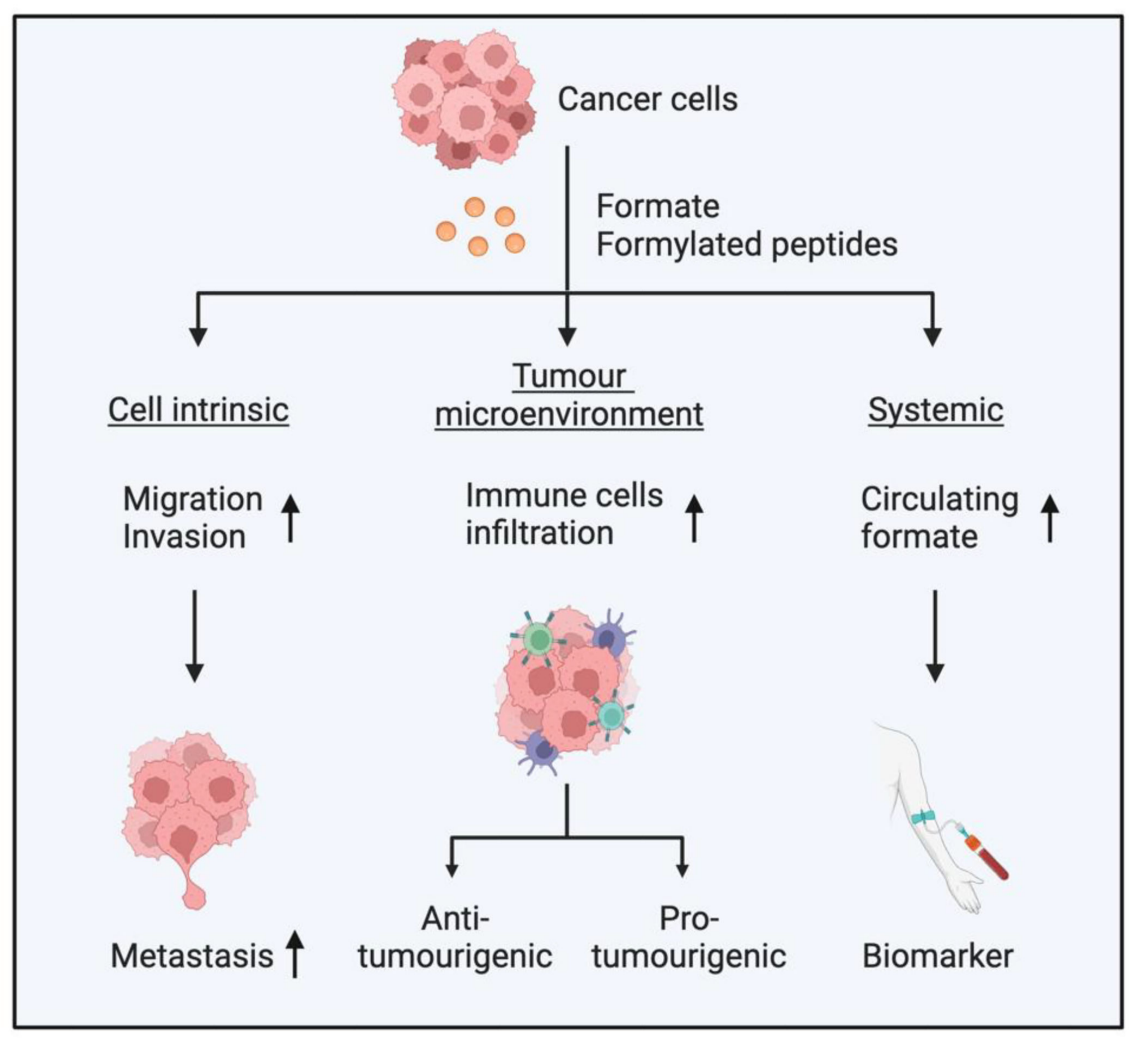
Potential consequences of formate metabolism in cancer. Cancer cells produce formate and formylated peptides, which can affect cancer cell-intrinsic properties, the tumour microenvironment (TME) and systemic metabolism. Formate increases migration and invasion of cancer cells, which leads to increased metastasis. Increased formate levels in the TME can promote infiltration of various immune cells, which can lead to pro- or anti-tumorigenic effects depending on the type of immune cells affected. Cancer cells-excreted formate can increase circulating formate levels, which have the potential to be used as a biomarker.

Serine and folate metabolism-derived compounds that are excreted by the cancer cells can have an impact on all the cells of the TME (Figure 5). Metabolites such as glycine and formate have been shown to accumulate in the TIF, with levels of formate potentially reaching up to 500μM in mouse models of breast cancer, compared to normal circulating formate levels of 30μM^101,129^. The induction of indoleamine 2,3-dioxygenase 1 (IDO1), a tryptophan degrading enzyme, in pancreatic ductal adenocarcinoma leads to increased formate overflow, which was shown to be utilized by surrounding pancreatic stellate cells for purine synthesis^30^. However, possibly the most important effect of cancer cell excreted 1C metabolites is the modulation of immune cells. Immune cells play a complex role in tumorigenesis, with different branches of the immune system showing pro- and anti-tumour activity^130^. Critical mediators of anti-tumour immunity are T-cells and while mitochondrial 1C metabolism is required for optimal T-cell activation, the field is just beginning to explore the effect of tumour derived metabolites such as formate or glycine on the immune population. A recent study showed that fusobacterium derived formate can contribute not only to colorectal cancer promotion but also Th17 T-cell expansion^34^. Furthermore, a role for formate in supporting T-cell activity and improving the responses to immune checkpoint inhibitors, such as anti-programmed cell death 1 (PD1), has also been reported^131,132^. As described above, silencing of ALDH1L2 in tumour cells leads to increased production of both formate and fMet, accompanied by enhanced signalling through the formyl-peptide receptor expressed by cancer cells. It is possible, therefore, that these tumour cell derived formylated peptides will also promote the migration of various immune cell populations and play a critical role in shaping the landscape of immune infiltration in tumours. Supporting this idea, overexpression of ALDH1L2 in cancer cells, decreasing the production of fMet, led to reduced numbers of neutrophils in a murine breast cancer models^101^. Taken together, these studies are beginning to reveal the complexity of the potential responses to tumour-derived formate, which may both promote tumour cell migration and enhance anti-tumour immunity. Overall, the effect of formate and fMet production by tumours on broader immune cell function – or even other cells of the TME - remains to be explored (Figure 5).

### Therapeutic approaches

The success of antifolates has driven attempts to target folate metabolism through different routes, with the aim of retaining therapeutic efficacy while reducing dose limiting toxicities. While the effectiveness of serine and folate cycle limitation can be explained by the restriction of nucleotide, NADPH and fMet production, other consequences of reduced serine availability that may contribute to the therapeutic effect have been described. These include an accumulation of toxic metabolites such as deoxysphingolipids and mitochondrial fragmentation/ceramide accumulation^105,133^.

Cancer associated changes that support malignant progression can also impose targetable vulnerabilities. For example, PHGDH overexpressing breast cancer cell lines require elevated NAD^+^ salvage pathway activity to meet the high demand for NAD^+^-dependent PHGDH activity. ER-negative, basal-like breast cancers show increased expression of both PHGDH and nicotinamide phosphoribosyltransferase (NAMPT), the rate-limiting enzyme of NAD^+^ salvage, and are sensitive to NAMPT inhibition^134^. While most cells use the mitochondrial folate cycle to capture 1C units, some cancer cells become dependent on cytosolic folate metabolism – a phenotype that has been linked to intracellular folate levels. These tumours become selectively sensitive to SHMT1 inhibition^135^. Moreover, PHGDH expression is required in breast cancer metastases – but not primary tumours – to allow α-KG-dependent mTORC1 signalling, making these cells sensitive to rapamycin, an mTORC1 inhibitor^113^. Increased serine metabolism leads to glycine accumulation and a dependence of cells on glycine removal systems such as the GCS – inhibition of which results in the production of toxic products such as aminoacetone and methylglyoxal^64^.

#### Dietary intervention

In mice, dietary limitation of serine has been shown to reduce circulating serine levels and have a therapeutic effect in multiple tumour models^49,124,136,137^. However, the response to this approach depends on various cancer cell intrinsic and extrinsic factors, including the tumour environment. Cancer cells with upregulated SSP activity, such as those driven by KRAS activation or those adapted to growth in serine depleted environments, are less likely to be affected by limitations of exogenous serine^49,124^. Dietary intervention may also be inadequate to effectively limit a high serine containing TME, or in situations where serine is provided by stromal cells. On the other hand, cancer cells with limited SSP activity could be highly responsive to dietary serine limitation. For example, this approach may be effective in luminal breast tumours, which tend to express low level of PSAT1 due to epigenetic silencing^137^, or the subset of pancreas cancer that show low PHGDH expression^125^. A subgroup of platinum resistant ovarian cancer showed decreased PHGDH expression at relapse – increasing the sensitivity of these tumours to serine free diets^138^ and suggesting this approach may become effective at later stages of tumorigenesis. p53-null cancers also show increased sensitivity to serine starvation and serine limiting diets^81^. Cancers harbouring Kelch-like ECH-associated protein 1 (KEAP1) mutations show enhanced NRF2-dependent antioxidant defence but become dependent on the uptake of several NEAA, including serine. In mouse models, KEAP1 mutations render tumours highly sensitive to a serine depleted diet^136^. Importantly, serine free diets can decrease metastases, even when primary tumour growth is only slightly affected^101^. Building on the successful impact of dietary serine depletion in mice, bespoke diets lacking a number of NEAA are now undergoing clinical trials to test for tolerability and efficacy in combination with standard of care chemotherapy in metastatic pancreatic cancer patients^139^.

#### Small molecule inhibitors

Inhibitors of several different steps of serine and folate metabolism have been developed and shown to be effective in preclinical models (Table 1). While several of these drugs were developed to inhibit different targets and were only subsequently shown to inhibit enzymes of the folate cycle (which may therefore be considered off-target effects), others were specifically designed to inhibit serine synthesis and folate metabolism. These include a number of PHGDH inhibitors, which are effective in limiting tumour growth in various preclinical models^43,140–142^. An SHMT/1/2 inhibitor, SHIN1, also showed efficacy in B-cell lymphoma and T-cell ALL models^143^, with evidence for a synergistic effect between SHIN2 and methotrexate^144^. Several MTHFD1 and MTHFD2 inhibitors have been developed and shown to limit tumour development^145,146^. One of these has a unique function in trapping folate and promoting the toxic accumulation of 10-formyl-THF^145^. Additionally, a study using artificial intelligence to predict collateral lethal metabolic pathways, revealed the targeting of MTHFD2 as a vulnerability for ovarian cancers lacking ubiquinol-cytochrome c reductase, complex III subunit XI (UQCR11)^147^. As noted for dietary approaches, use of these inhibitors will be limited by the metabolic landscape of the cancer. Tumours growing in serine limited environments – such as the mammary gland – are dependent on de novo serine production^124^ and tumours that metastasise to the brain – another serine limited environment – are highly responsive to treatment with a PHGDH inhibitor^148^. Another interesting approach is to inhibit GCPII, thereby reducing the folate pools available to cancer cells. Prostate cancers frequently show upregulation of GCPII, leading to an exploration of the efficacy of GCPII inhibition as a therapeutic option in these tumours^149^.

Currently, we are not aware of ongoing clinical trials of drugs that have been designed to target serine or folate metabolism. It is possible that their use will be limited by on-target toxicities resulting from the systemic inhibition of these pathways or by difficulty in achieving sufficient impact on highly expressed metabolic enzymes. Some of the drugs listed in Table 1 that were designed to hit other targets have been clinically tested, but their pleiotropic functions make it difficult to assess how much of their impact is due to the inhibition of the folate cycle enzymes.

#### Effect on stromal cells

Dietary serine starvation or treatment with folate cycle inhibitors will have systemic impacts on normal, as well as tumour cells - especially cells with high proliferation rates such as blood, gut or activated immune cells. While mice were shown to tolerate serine starvation in the short-term, long-term maintenance on a serine free diet led to peripheral neuropathy, which was accelerated by high fat intake^179^. T-cells, which are critical mediators of anti-tumour immunity are highly dependent on serine and likely to be compromised by serine-limiting therapies. Indeed, dietary restriction of serine can impair T-cell expansion upon pathogen infection^180^ while serine synthesis and mitochondrial 1C metabolism is necessary for T-cell survival and antigen specific T-cell abundance *in vivo*^181,182^. Perturbation of folate metabolism by depletion of MTHFD2 promotes the differentiation of regulatory T-cells (Tregs), which can have tumour supportive function^183^. Consistently, increased serine metabolism in Tregs can restrict their function, a response that was rescued by limiting serine^184^. Defective 1C metabolism is observed in aged mice, contributing to a compromised T-cell response and consistent with the age associated increase in cancer burden^107^. Serine synthesis is also important in macrophages^185^, where high levels of PHGDH expression drives the polarization of these cells towards an immunosuppressive and cancer supporting phenotype^186^. The SSP is also required for germinal centre formation and antibody production, with overexpression of PHGDH necessary to allow the survival of large B-cell lymphoma^142^. Similarly, while formate production may be an attractive therapeutic target to limit metastatic potential of cancer cells, the role of formate and formylated peptides in provoking an immune response raises the possibility that such approaches could also blunt anti-cancer immunity.

Taken together, it will be critical to assess whether systemic serine metabolism modulating therapies dampen an effective anti-tumour immune response.

#### Combinational therapy

Dietary modulation will only reduce – but not eliminate – the availability of NEAA to fuel folate metabolism and so is unlikely to be effective as a monotherapy. However, approaches that combine specific diets with matched small molecule inhibitors have shown promise in preclinical trials^187,188^. Serine free diets combined with a PHGDH inhibitor showed enhanced anti-tumour activity in mice, although this combination therapy also led to dose limiting weight loss, most likely reflecting a systemic drop in serine that was not apparent with either treatment alone^43^. As mentioned earlier, serine starvation may also help to overcome 5-FU resistance^92^. Intriguingly, although histidine can be a source of 1C units, histidine catabolism in cancer cells led to the production of 5-formyl-THF and the depletion of the intracellular THF pool – resulting in enhanced response to methotrexate in a leukaemia xenograft model^189^.

Lack of serine leads to increased intracellular ROS, due to a reduction in components of antioxidant defence mechanisms and has been shown to sensitize tumours to ROS-inducing radiotherapy^190^. Similarly, serine depletion synergises with biguanides such as phenformin or metformin to limit cancer growth in mice^49,191^. While this response may reflect the activity of biguanides in limiting mitochondrial activity and increasing ROS, the ability of metformin to directly inhibit SHMT2 suggests another mechanism by which this drug can enhance the effectiveness of serine limitation^168^.

Cancer therapy has been transformed over the past ten years by the availability of immune checkpoint inhibitors that target PD1 or PD1 ligand 1 (PDL1). These drugs release the brake on T-cell activation imposed by cancer cells and restore an effective anti-tumour immune response^192^. As discussed above, serine and folate metabolism are important for the activation of effective T-cell responses, so it is surprising and encouraging that pemetrexed – an antifolate that is standard of care for non-small cell lung cancer patients – was shown to synergise with anti-PD1^193^. PSPH was shown to be overexpressed in HCC, promoting the SSP and modulating the immune response to these tumours by attracting tumour supporting monocytes/macrophages while limiting T-cell recruitment. Inhibition of PSPH reversed these immune-limiting effects, a response that was enhanced by anti-PD1 treatment^194^. Intriguingly, metformin was shown to inhibit PSPH in this study, suggesting another potential mechanism for biguanide function. A beneficial effect of formate supplementation in combination with checkpoint inhibitors on CD8+ T cell antitumor activity suggests that in some contexts, enhanced folate cycle activity in immune or tumour cells may be beneficial for cancer therapy^131,132^.

Checkpoint inhibition has curative potential, but many patients fail to respond to these therapies. While the efficacy of combinations with folate pathway inhibitors remains to be fully validated in clinical trials, the data support a broader view that understanding the metabolic requirements and vulnerabilities of cancer cells will allow for the development of targeted therapeutic options to exploit these cancer specific changes. The concept of specific dietary modulation matched to tumour type, oncogenic changes and location (precision nutrition) is especially attractive to cancer patients who frequently search for diets that will help improve their survival^187^.

## Future Directions

Early success in modulating folate metabolism in cancer therapy has suggested that additional therapeutic targets may be identified in these pathways. Preclinical studies are revealing numerous potential points of intervention and effective combinatorial approaches, although most of these await clinical validation. Several considerations may be pertinent to future success in this area. Firstly, the complexities of how different cell types in different environments show differences in their requirements and use of various metabolites will need careful assessment. As an example, the production of mitochondrial NADPH has been shown to play a key role in limiting ROS (as described here), but in some cell systems – where mitochondrial NADPH can be obtained from alternative sources – 1C cycle derived NADPH is more important in supporting lipogenesis or proline synthesis^20,93,95,103,104^. The plasticity of metabolism may also represent a barrier to the development of effective therapeutics. However, metabolic plasticity may be more evident in nutrient rich tissue culture conditions than in vivo, where nutrient limitation may restrict the available options for metabolic rewiring. Indeed, much of the work in this area has been based on the response of cancer cells grown in tissue culture and while these studies have been successful, the use of more physiologically accurate conditions – such as culture media that contain physiological levels of various nutrients - may more effectively point to targetable vulnerabilities^129,195^. In support of this suggestion, the dependence of many cancer cells on exogenous serine was to some degree mitigated in physiological media due to the presence of hypoxanthine to support nucleotide synthesis^196^. Future use of media reflecting nutrient composition of TIFs or organ sites of various cancers and metastases, coupled with analysis of organoids – which are more reflective of the architecture of cancers *in vivo* and can accommodate several cell types to mimic at least some aspects of tumour/stromal interactions - may help to identify new therapeutic options. Interestingly, up to 500μM of formate has been measured in TIF of 4T1 breast cancers compared to 10-50μM in plasma, and circulating formate levels were shown to be increased in different genetically engineered mouse models of cancer^109^. It is therefore possible that increased formate could be used as a circulating biomarker for early cancer detection or monitoring of therapeutic response, although detection of both increased and decreased circulating formate levels in cancer patients^110^ shows that further work is needed to establish such an approach. One limitation at present is the requirement for robust systems of metabolite detection. The detection of formate – for example – is not usually part of a routine metabolomics analysis and the failure to measure certain metabolites will lead to an underestimation of the role they play. However, great strides have been made and while the therapeutic success of metabolic interventions has to date been limited, we are optimistic that an increased understanding of metabolic demands, vulnerabilities and plasticity of cancer cells will soon lead to improved therapy.

## Figures and Tables

**Table 1:** Drugs targeting serine and one-carbon metabolism in cancers.

Agent	Target (s)	Status	Reference
Azacoccone E	PHGDH	Preclinical	^150^
BI-4916/4924	PHGDH	Preclinical	^151^
CBR-5884	PHGDH	Preclinical	^152^
Disulfiram	PHGDH	FDA-approved to treat alcohol dependence	^153^
Ixocarpalactone A	PHGDH	Preclinical	^154^
NCT-502/503	PHGDH	Preclinical	^140^
Oridonin	PHGDH	Preclinical	^155^
PH-739/755	PHGDH	Preclinical	^43,148^
PKUMDL-WQ-2101	PHGDH	Preclinical	^141^
Compound C25	PHGDH	Preclinical	^156^
Compound D8	PHGDH	Preclinical	^157^
Thimerosal	PHGDH	Preclinical	^158^
Withangulatin A	PHGDH	Preclinical	^159^
Chlopromazine	PSPH	FDA-approved to treat schizophrenia, bipolar disorder and acute psychosis	^160^
Clofazimine	PSPH	FDA-approved to treat leprosy and tuberculosis	^161^
CMPSA	PSPH	Preclinical	^162^
Glycerophosphorylcholine	PSPH	Clinical trial for dementia and physical and psychomotor performance	^162^
Jung11	PSPH	Preclinical	^161^
L-AP3 D-AP3	PSPH	Preclinical	^163^
AGF291 AGF320 AGF347	SHMT1/2	Preclinical	^164^
AM-807/42004511 AM-807/40675298 AM-807/42004633	SHMT2	Preclinical	^165^
Compound 2.12	SHMT1	Preclinical	^166^
Lometrexol, pemetrexed	SHMT2	Lometrexol (clinical trial), pemetrexed (FDA-approved as chemotherapy)	^167^
Metformin	SHMT2	FDA-approved to treat type 2 diabetes mellitus	^168^
RZ-2994	SHMT1/2	Preclinical	^169^
Sertraline	SHMT1/2	FDA-approved as antidepressant	^158^
SHIN1	SHMT1/2	Preclinical	^143^
SHIN2	SHMT1/2	Preclinical	^144^
3-bromopyruvate	SHMT1/2	Preclinical	^170^
Carolacton	MTHFD1/2	Preclinical	^171^
Compound 1, 2, 3, 4R, 4S, 5	MTHFD2	Preclinical	^172^
C18	MTHFD2	Preclinical	^173^
DS18561882	MTHFD2	Preclinical	^146^
DS44960156	MTHFD2	Preclinical	^174^
LY345899	MTHFD1/2	Preclinical	^175^
TH7299 TH9028 TH9619	MTHFD1/2/2L	Preclinical	^176,177^
Xanthine derivative 15	MTHFD2	Preclinical	^178^
Standardized Nonessential Amino Acid Restriction	Nonessential Amino Acid	Clinical trial	^139^
